# Acute appendicitis revealing a diagnosis of chronic myelogenous leukemia

**DOI:** 10.1002/ccr3.3902

**Published:** 2021-02-24

**Authors:** Rita Ahmad, Elrazi Ali, Lina Okar, Orwa Elaiwy, Mohamed Abdelrazek, Yahya Mulikandathil, Mohamed Yassin

**Affiliations:** ^1^ Department of Family Medicine Hamad Medical Corporation Doha Qatar; ^2^ Department of Internal Medicine Hamad General Hospital Hamad Medical Corporation Doha Qatar; ^3^ Department of Pathology Hamad Medical Corporation Doha Qatar; ^4^ Department of Radiology Hamad Medical Corporation Doha Qatar; ^5^ Department of Hematology/Oncology National Centre for Cancer Care & Research Doha Qatar

**Keywords:** appendicitis, typhlitis, chronic myelogenous leukemia, leukemia, chronic myelogenous leukemia, hematologic malignancies

## Abstract

Gastrointestinal manifestations of leukemias have been well recognized. Typically, acute leukemias cause typhlitis or appendicitis more commonly than chronic leukemias. Our case points to appendicitis as possible manifestation of chronic myelogenous leukemia.

## Graphical abstract

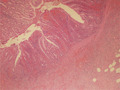

## 1 INTRODUCTION

Hematologic malignancies are becoming more prevalent, lymphomas and leukemias, manifesting in various clinical scenarios. Chronic myelogenous leukemia (CML) is one of the myeloproliferative neoplasms that is usually diagnosed accidentally on routine complete blood count (CBC) because it is asymptomatic in most cases. However, it can cause multiple signs and symptoms and affect many organs, including the gastrointestinal system. We present a case of a 36‐year‐old patient presented with appendicitis found to have CML during his workup for his abdominal pain.

Hematologic malignancies (HM) represent a burden on the healthcare system worldwide, mainly due to their prevalence among the pediatric population, and have always been well recognized among other types of cancer by the effective use of genetic analyses to establish the diagnosis, classification, and prognosis.^1^ HM include leukemias, lymphomas, myeloproliferative neoplasms (MPNs), mast cell neoplasms, plasma cell neoplasms, histiocytic tumors, and dendritic cell neoplasms. However, leukemias and lymphomas comprise the majority of malignant cases, with acute lymphoblastic leukemia being the most common malignancy in children.^2^ Chronic leukemias include lymphocytic and myelogenous leukemias, which carries an overall more benign course than acute leukemias and occurs typically in the adult population.^3^ Chronic myeloid/ myelogenous leukemia (CML) is one of the MPNs and accounts for 15% of newly diagnosed leukemia in adults; it is characterized by the presence of BCR‐ABL1 fusion oncogene; this rearrangement is known as the Philadelphia chromosome.^4^ CML can range from an asymptomatic course, which accounts for almost 50% of cases diagnosed in the United States, to a wide array of signs and symptoms, including fatigue, weight loss, fever, abdominal discomfort, and many other manifestations.^4^ Among the various presentations, gastrointestinal (GI) involvement has been well recognized, though it is more common in acute leukemias than chronic, and is becoming even less common due to improved chemotherapy.^5^

Here, we present a case of a 36‐year‐old male who presented with right lower abdominal pain and was accidentally found to have a very high white blood cell (WBC) count during routine workup 317.3 × 10^9^/L (4‐10 × 10^9^/L).

## 2 CASE PRESENTATION

Thirty‐six years old Indian gentleman with no previous medical or surgical history presented to the emergency department complaining of diffuse abdominal pain for 1 day. The pain was associated with repeated vomiting and loss of appetite. Initially, the pain was generalized and vague, and then became more localized to the right lower abdomen. There were no hematemesis or melena, and no history of fever or upper respiratory symptoms. No dysuria or change in bowel habits. No scrotal or testicular pain. On physical examination, there was tenderness in the right lower quadrant. Blood investigations showed significantly elevated WBC 317.3 × 10^9^/L (4‐10 × 10^9^/L) (Laboratory results in Table 1).

**TABLE 1:** Laboratory tests and results

Laboratories	Parameter	Normal value
WBC	317.3 × 10^3^/μL	4‐10 × 10^3^/μL
PLT	146 × 10^3^/μL	150‐400 × 10^3^/μL
Hb	10.7 g/dL	13‐17 g/dL
Absolute neutrophil count	184 × 10^3^/μL	2‐7 × 10^3^/μL
Eosinophil	3.2 × 10^3^/μL	0.0‐0.5 × 10^3^/μL
Basophil	6.35 × 10^3^/μL	0.02 × 0.1 × 10^3^/μL
Lymphocytes	3.2	1‐3 × 10^3^/μL
RBC folate	2110 nmol/L	1187‐2854 nmol/L
B12	1024.0 pmol/L	125‐569 mol/L

**FIGURE 1:**
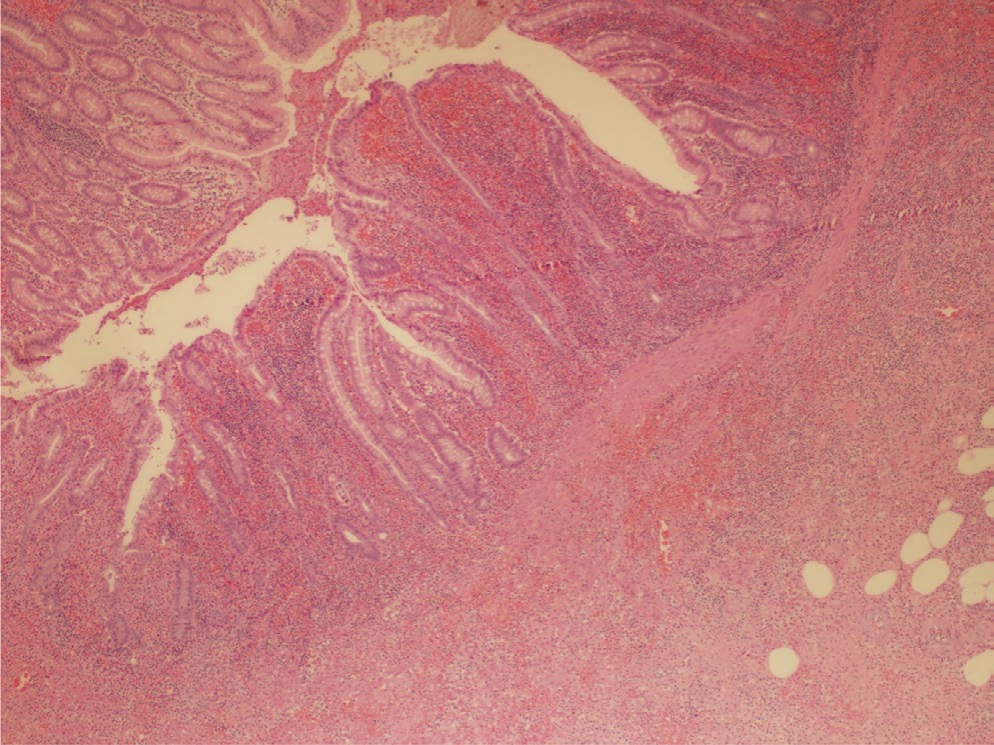
Picture 40×: Representative section from the appendix wall showing hemorrhage with acute transmural inflammation (Hematoxylin and eosin stain, 40×)

**FIGURE 2:**
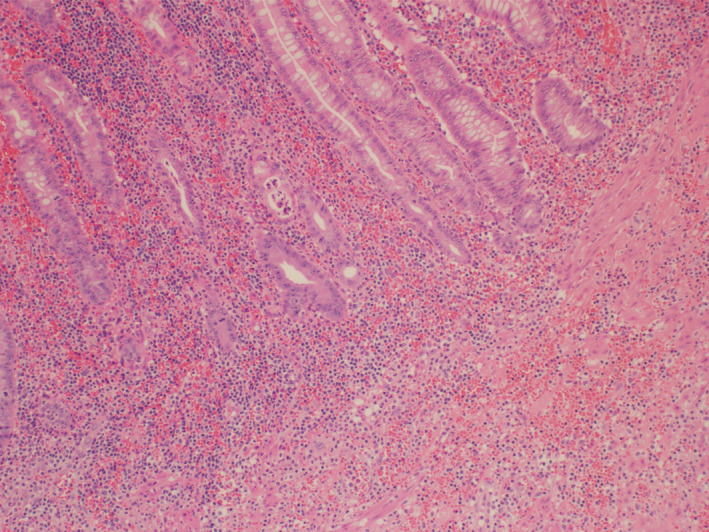
Picture 100×: Representative section from the appendix wall showing hemorrhage with acute transmural inflammation. Some glands are invaded by neutrophils in the center and left aspect of the image (Hematoxylin and eosin stain, 100×)

CT scan of the abdomen (radiology images A and B) showed moderate splenomegaly and dilated appendix, measuring 1.8 cm in maximum diameter with periappendiceal fat stranding and minimal free fluid in the right iliac fossa. No appendicoliths are noted, suggestive of acute appendicitis. He went for laparoscopic appendectomy and was found to have acute gangrenous appendicitis intraoperatively. The appendix was sent for histopathology, which revealed severe acute appendicitis (Figures 1 and 2).

After surgery, further investigation for leukocytosis was done. Peripheral smear showed normochromic normocytic anemia with severe leukocytosis, shift to left, and basophilia, suggestive of a myeloproliferative disorder. Cytogenetic and molecular studies were positive for an e14a2 BCR‐ABL1 gene fusion by single‐step RT‐PCR. No evidence of mutations within the JAK2 or CALR genes. Interphase fluorescence in situ hybridization (FISH) showed BCR/ABL1 rearrangement in 97% of nuclei. Bone marrow biopsy showed hypercellular bone marrow (~90%‐100% cellularity) with remarkable granulocytic hyperplasia with only 2% blast. Karyotype result showed 46,XY,*t*(9;22)(q34; q11.2).

The patient was started on hydroxyurea 1 g three times daily and rasburicase. After confirming the diagnosis, the patient preferred to get treatment in his home country and traveled back to India.

## 3 DISCUSSION

Leukemias constitute one overwhelming branch of the hematologic malignancies and are mainly divided into lymphoblastic/lymphocytic and myeloblastic/ myelogenous leukemias. CML is one of the MPNs and accounts for 15% of diagnosed cases of leukemia in adults.^6^ It is characterized by the translocation *t*(9;22)(q34;q11.2), resulting in the fusion of the Abelson murine leukemia (*ABL1*) gene on chromosome 9 with the breakpoint cluster region (*BCR*) gene on chromosome 22 (BCR‐ABL1), and this rearrangement is known as the Philadelphia chromosome.^4^, ^6^, ^7^ Most cases of CML are asymptomatic, and the diagnosis is made via a routine complete blood count (CBC), while symptomatic patients may have fatigue, fever, weight loss, anemia, and abdominal discomfort due to splenomegaly.^5^ Generally, CML can be classified into three phases: chronic phase (CP), accelerated phase (AP), and blast phase (BP).^4^ The majority of patients present with CP. GI manifestations of CML are well established in the literature, occurring more commonly in acute leukemias than chronic.^5^ Reported autopsies showed that the GI tract involvement in leukemias is seen in about 25%.^5^ Previous studies from the 1960s have described GI involvement in the context of leukemias, stating that the stomach is the most common site of involvement.^8^ This was further emphasized in recent literature that the most common sites are the stomach, ileum, and proximal colon.^5^ These leukemic lesions invade mucosa and submucosa and may complicate in ulceration or perforation. Leukemic lesions of the esophagus can be hemorrhagic, ranging from mild petechiae to ulcers and erosions, or can be infiltrative and may cause obstruction or perforation.^5^ Leukemic infiltration of the lymphoreticular system, particularly liver, spleen, and lymph nodes, is quite common, especially in chronic leukemias. Significant splenomegaly is commonly seen in CML, and to a lesser extent, in chronic lymphocytic leukemia (CLL) and acute leukemias; however, splenic rupture with no history of trauma was recorded in acute leukemias.^5^ When involving the small and large intestines, leukemic lesions lead to what is called neutropenic enterocolitis or necrotizing colitis, clinically known as typhlitis, which can be hard to distinguish from appendicitis as the case in our patient who was initially suspected to have typhlitis when the CML diagnosis was made. However, typhlitis occurs more frequently with acute leukemias as a complication of neutropenia, which is a cardinal feature, but when typhlitis occurs in chronic leukemias is typically due to chemotherapy and the neutropenia that follows.^5^, ^9^ Distinguishing between appendicitis and typhlitis is crucial in leukemic patients as the management is completely different. Wallace et al described a case of properly diagnosed appendicitis in a 24‐year‐old CML patient, which occurred 3 years after being diagnosed, being treated with multiple‐agent chemotherapy regimen after the presence of circulating blasts, and finally underwent bone marrow transplant (BMT). The patient was in relapse during his admission for an acute abdomen, which was confirmed to be appendicitis.^9^ However, our patient presented with abdominal pain, which was found to be due to appendicitis before even establishing a diagnosis of CML. There is paucity in the literature regarding CML per se and GI manifestations. On the other hand, several studies have described the effect of CML treatment on GI tract and other organs.^10^, ^11^ The oncogene BCR‐ABL1 leads to activation of tyrosine kinase, so the mainstay of treatment is tyrosine kinase inhibitors (TKIs) such as imatinib, and more recently, the second‐generation TKIs, dasatinib, and nilotinib.^6^ Agents are used first line in the treatment of CML and have been very effective in achieving complete cytogenetic response (CCR); however, their adverse events should be considered when initiating treatment and when involving patients with their disease treatment and plan.^11^ Several case reports described such adverse events, and Yassin et al described a case of hemorrhagic colitis in a patient with CML who was treated with dasatinib 100 mg once daily and achieved CCR after 30 months of treatment; however, she complained of watery and bloody diarrhea, she underwent colonoscopy with biopsies that confirmed the diagnosis of cytomegalovirus (CMV) colitis after excluding other possible causes such as clostridium difficile, dasatinib was discontinued, and the patient improved spontaneously within 5 days. The patient was treated with ganciclovir for 6 weeks, and the follow‐up colonoscopy showed a normal mucosa.^11^, ^12^ Another case report in 2018 by Nacif et al reported a case of imatinib‐induced fulminant hepatitis in a patient treated for CML, and the patient had a successful live transplant and was started on dasatinib afterward with no adverse effects.^10^

In conclusion, leukemias whether acute or chronic may manifest in a variety of symptoms and conditions, and patients should be addressed with caution and in a comprehensive approach.

## CONFLICT OF INTEREST

None declared.

## AUTHOR CONTRIBUTION

RA: manuscript writing and literature review. EA: case presentation writing. LO: literature review. OE: pathology slides. MA: radiology slides. YM: literature review. MY: mentorship, manuscript writing, and literature review.

## ETHICAL APPROVAL

Ethical Approval was obtained by Medical Research Center (MRC) under ID MRC‐04‐20‐977 on October 27th, 2020. The article processing charges were funded by Qatar National Library (QNL).

## Data Availability

All data generated during this study are included in this article.

## Appendix 1

### Radiology Images A and B

Axial (A) and coronal (B) computed tomography of the abdomen without oral or intravenous contrast administration show dilated appendix (yellow arrow in A & B) measuring about 18 mm in maximum anteroposterior diameter with mild periappendiceal fat stranding. No detected appendicolith.

## References

[1] Taylor J , Xiao W , Abdel‐Wahab O . Diagnosis and classification of hematologic malignancies on the basis of genetics. Blood. 2017;130(4):410‐423. 28600336 10.1182/blood-2017-02-734541 PMC5533199

[2] Ward E , DeSantis C , Robbins A , Kohler B , Jemal A . Childhood and adolescent cancer statistics, 2014. CA Cancer J Clin. 2014;64(2):83‐103. 24488779 10.3322/caac.21219

[3] Yassin MA , Taher A , Mathews V , et al. MERGE: a multinational, multicenter observational registry for myeloproliferative neoplasms in Asia, including Middle East, Turkey, and Algeria. Cancer Med. 2020;9:4512‐4526. 32351024 10.1002/cam4.3004 PMC7333830

[4] Yassin MA , Abdulla MA , Chandra P , et al. Chronic myeloid leukemia in adolescents and young adults: a single institute experience. Blood. 2019;134:5915.

[5] Ebert EC , Hagspiel KD . Gastrointestinal manifestations of leukemia. J Gastroenterol Hepatol. 2012;27(3):458‐463. 21913980 10.1111/j.1440-1746.2011.06908.x

[6] Turkina A , Wang J , Mathews V , et al. TARGET: a survey of real‐world management of chronic myeloid leukaemia across 33 countries. Br J Haematol. 2020;190:869‐876. 32227648 10.1111/bjh.16599

[7] Swerdlow SH , Campo E , Pileri SA , et al. The 2016 revision of the World Health Organization classification of lymphoid neoplasms. Blood. 2016;127(20):2375‐2390. 26980727 10.1182/blood-2016-01-643569 PMC4874220

[8] Cornes JS , Jones TG . Leukaemic lesions of the gastrointestinal tract. J Clin Pathol. 1962;15:305‐313. 13881389 10.1136/jcp.15.4.305 PMC480404

[9] Wallace J , Schwaitzberg S , Miller K . Sometimes it really is appendicitis: case of a CML patient with acute appendicitis. Ann Hematol. 1998;77(1‐2):61‐64. 9760155 10.1007/s002770050413

[10] Nacif LS , Waisberg DR , Pinheiro RS , et al. Imatinib‐induced fulminant liver failure in chronic myeloid leukemia: role of liver transplant and second‐generation tyrosine kinase inhibitors: a case report. J Med Case Reports. 2018;12(1):63. 10.1186/s13256-018-1588-0 PMC5845231 29523185

[11] Yassin MA , Nashwan AJ , Soliman AT , et al. Cytomegalovirus‐induced hemorrhagic colitis in a patient with chronic myeloid leukemia (chronic phase) on dasatinib as an upfront therapy. Clin Med Insights Case Rep. 2015;8:77‐81. 26379451 10.4137/CCRep.S25327 PMC4554353

[12] Riaz LM , Galal KM , Hussein RM , Nashwan AJ , Yassin MA . Dasatinib‐induced colitis in a patient with chronic myeloid leukemia (chronic phase). Int J Case Rep. 2018;3:43. 10.28933/ijcr-2018-10-240

